# A combination of fuzzy Delphi method and hybrid ANN-based systems to forecast ground vibration resulting from blasting

**DOI:** 10.1038/s41598-020-76569-2

**Published:** 2020-11-10

**Authors:** Jiandong Huang, Mohammadreza Koopialipoor, Danial Jahed Armaghani

**Affiliations:** 1 School of Mines, China University of Mining and Technology, Xuzhou, 210006 China; 2 Faculty of Civil and Environmental Engineering, Amirkabir University of Technology, 15914 Tehran, Iran; 3 Institute of Research and Development, Duy Tan University, Da Nang, 550000 Vietnam

**Keywords:** Environmental impact, Geology, Civil engineering

## Abstract

This study presents a new input parameter selection and modeling procedure in order to control and predict peak particle velocity (PPV) values induced by mine blasting. The first part of this study was performed through the use of fuzzy Delphi method (FDM) to identify the key input variables with the deepest influence on PPV based on the experts’ opinions. Then, in the second part, the most effective parameters on PPV were selected to be applied in hybrid artificial neural network (ANN)-based models i.e., genetic algorithm (GA)-ANN, particle swarm optimization (PSO)-ANN, imperialism competitive algorithm (ICA)-ANN, artificial bee colony (ABC)-ANN and firefly algorithm (FA)-ANN for the prediction of PPV. Many hybrid ANN-based models were constructed according to the most influential parameters of GA, PSO, ICA, ABC and FA optimization techniques and 5 hybrid ANN-based models were proposed to predict PPVs induced by blasting. Through simple ranking technique, the best hybrid model was selected. The obtained results revealed that the FA-ANN model is able to offer higher accuracy level for PPV prediction compared to other implemented hybrid models. Coefficient of determination (R^2^) results of (0.8831, 0.8995, 0.9043, 0.9095 and 0.9133) and (0.8657, 0.8749, 0.8850, 0.9094 and 0.9097) were obtained for train and test stages of GA-ANN, PSO-ANN, ICA-ANN, ABC-ANN and FA-ANN, respectively. The results showed that all hybrid models can be used to solve PPV problem, however, when the highest prediction performance is needed, the hybrid FA-ANN model would be the best choice.

## Introduction

Nowadays, blasting is recognized as the most cost-effective technique that cane efficiently applied to fragmentation of rock mass in tunneling, surface mine, and construction operations^1^. Blasting is mainly conducted to achieve a proper fragmentation of rock mass in order to make easier the loading and transportation operations. On the other hand, blasting also brings about some unwanted environmental results such as ground vibration, flyrock, and air-overpressure^2–5^. This is why there is a pressing need for designing models that can effectively and accurately evaluate and predict such detrimental effects, which can finally result in enhancing the safety level of blasting projects. Among the mentioned environmental issues, blast-induced ground vibration (BIGV) is one of the most undesirable effects and it can make vibration of the structures; cause instability of mine’s slopes; and puzzling for neighboring communities^6,7^. Several reports have been released indicating the concerns of people living in the vicinity of open-pit mines regarding their safety and health issues. As result, this is of a high importance and interest to provide a precise prediction technique of BIGV for controlling and minimizing this parameter before blasting operations^8^.

**Figure 1:**
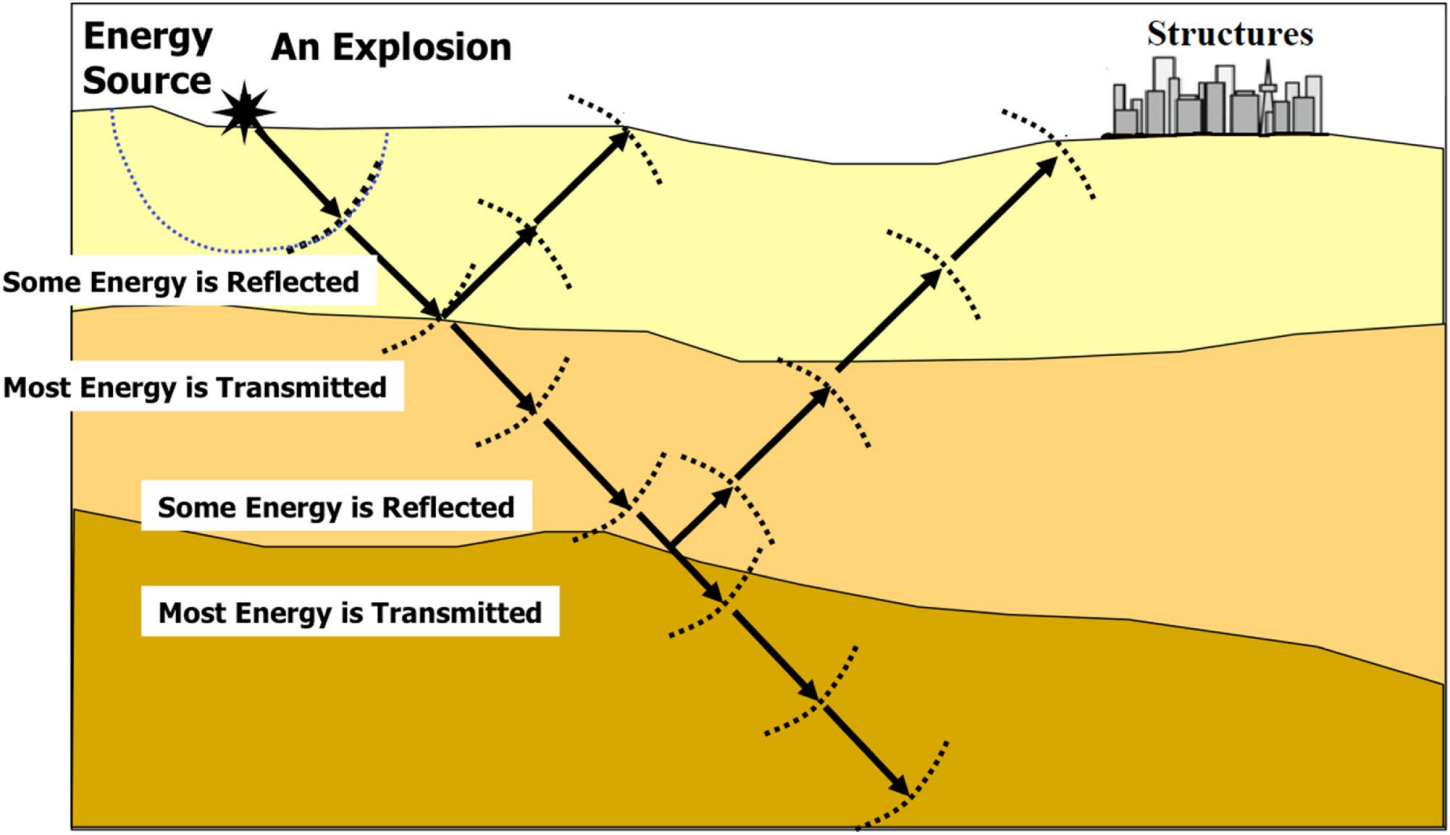
Ground vibration induced by blasting.

BIGV refers to wavy motions starting in the blasting site and travelling away to adjacent areas^7^. In each BIGV, a certain quantity of energy is spent, which is assumed to be used in fracturing the rock. This phenomenon significantly affects the ecology, groundwater, and constructions that exist in the neighborhood^9^. After explosion within blast hole, high-temperature gas and a high-pressure will be created, which are chemical reactions of the explosive materials. Such pressure is able to crush the rock nearby the blast hole. The pressure induced by the explosion will rapidly decay or dissipate. A wavy motion is shaped within the ground by the strain waves, which reaches the neighboring rocks^10^. The gases with high temperature and high pressure spread out the radial cracks, fractures, discontinuities, or joints^11^. The strain waves spread just like the elastic ones at the time the strength of the stress wave reduces to an extent in which there is not risk of any enduring deformation to take place within rock mass (Fig. 1). Such waves are normally known as the ground vibration that is propagate starting from the blast hole towards all directions around^11^.

In BIGV, there are lots of parameters that have significant effects on this process, e.g., the geological settings, the design of blasting, and the distance between monitoring point and free face (blasting face)^12^. The parameters of blasting design need to be optimized in a way to minimize the ground vibrations, and this should be done according to rock mass properties such as strength, density, discontinuity conditions, and velocity of the waves produced. The values of BIGV are measurable regarding both frequency and the peak particle velocity (PPV). The PPV is a vibration index significantly indicating the control of the physical damages^13^. Because of the ground vibrations that normally take place at the time of surface blasting, PPV can be considered as a great index for prediction of such vibrations. Two different parameters have significant impact on PPV, i.e., the distance from the free face and the maximum charge per delay^14^.

The PPV phenomenon was first studied experimentally by various researchers^9,13,15^. However, these experimental equations have two major weaknesses: (1) they receive a low rate of accuracy and (2) they are site specific^16^. One of the main reasons for these weaknesses is that they used a limited number of influential factors on the PPV^17^. Numerous studies confirmed that the empirical equations may lose their efficiency when used in the other sites^18^. In addition to the empirical equations, statistical techniques were also used by the designers. As a result, they also have weakness in PPV evaluation due to their low flexibility against new used parameters^19^.

To overcome the weaknesses of previous models, it is necessary to use a new system that can solve problems with different conditions. Artificial intelligence (AI)-based models which consider as new approaches, are able to solve non-linear problems. Due to the widespread use of the AI models in the field of mining and civil engineering^20–49^, various AI models were used for more accurate prediction and evaluation of PPV induced by blasting in this research. Many researchers have studied engineering topics using basic AI models such as artificial neural network (ANN), fuzzy system and neuro-fuzzy^50,51^. Others have offered various extensions to implement optimization algorithms and combine them with predictive models^52,53^.

ANNs networks can be effectively applied to problems related to prediction issues, and at the same time, they suffer from a number of limitations like falling into local minima and having a low speed of learning^54^. Literature^55–57^ pointed out that such drawbacks can be overcome through the use of some effective optimization algorithms (OAs) such as imperialism competitive algorithm (ICA), genetic algorithm (GA), artificial bee colony (ABC), firefly algorithm (FA), and particle swarm optimization (PSO). High competence of global search in the above-mentioned OA algorithms helps to determine the biases and weights of the ANN system in a way to enhance its efficiency and accuracy level of predictive models.

Aiming at the prediction of PPV, the present study tries to develop five hybrid ANN-based models, i.e., ICA-ANN, GA-ANN, ABC-ANN, FA-ANN and PSO-ANN. The models were suggested based on the key parameters that affected PPV according to experience of several experts in field of blasting. Fuzzy Delphi Method (FDM) was applied to the identification of the key variables that have influence on the results obtained from PPV. In definition, FDM refers to an approach on that basis of the experts’ opinions; this have been shown as a reliable instrument to obtain valuable knowledge in regard to the significance of the variables. In fact, in this research, the model inputs have been selected by FDM and then used in modeling of PPV prediction. In the rest of the study, the predictive models considered here are introduced; the established database are elaborated; next, the modelling processes of the adopted techniques will be fully described; and finally, the model with the highest competency in predicting PPV will be chosen and introduced.

## Principles of the predictive models

### Artificial neural network (ANN)

In general, artificial neural networks (ANNs) make available a bio-inspired computational model capable of mimicking the mechanisms of human being’s brain in logical reasoning. According to the problem, the components (i.e., connection pattern, learning rule and activation function) are determined in a way to train the system through the adjustment of its weights^58^. Multilayer perceptron (MLP), which is a commonly-used feedforward neural network (FFNN), comprises an input layer of source nodes, an output layer, as well as a minimum number of 1 as hidden layer. In a FFNN, first, the hidden neurons (nodes) process the flowing signals to specify the underlying features of input patterns. After that, the specified features are transferred to the output nodes for the purpose of extracting the output pattern^59^.

In recent decades, a variety of learning techniques have been introduced to literature for the improvement of the learning capacity of the MLP networks. Backpropagation (BP), among all, has shown the highest level of attractiveness to the researchers of the field an efficient gradient descent-based method^60–62^. In the course of each epoch in BP, one output is generated by exchanging the input signals amongst the computational nodes of succeeding layers. Equation (1) is used to calculate the net weighted input *net*_*j*_ fed to each node:

1 $$net_{j} = \sum\limits_{i = 1}^{n} {x_{i} w_{i}^{{ - {\uptheta }}} }$$

where *n* stands for the number of inputs, *w*_i_ and *x*_*i*_ signify weight and input signal for the *i*th node, respectively, and *θ* represents the threshold applied to the nodes. An activation function (e.g., step, sigmoid, or linear functions) is used to pass this net input. In a technical view, such procedure is termed training procedure. Then, to compute the output error, a comparison is made between the predicted output and the actual one^63^. At the final step (i.e., the backward pass step), through the network, the resulting error is flown back aiming at fine-tuning the individual weights.

### Particle swarm optimization (PSO)

**Figure 2:**
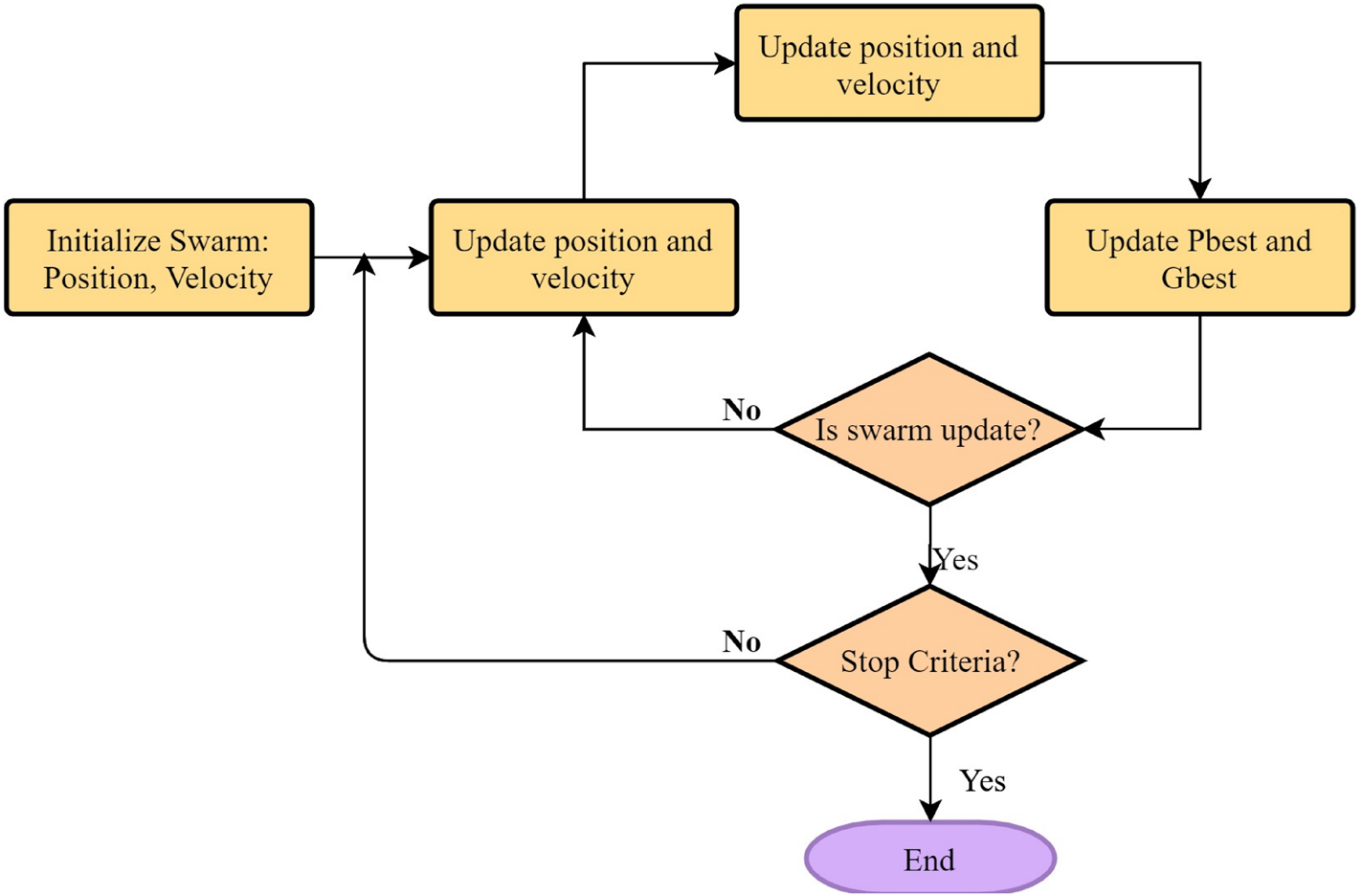
A flowchart of PSO algorithm.

Particle swarm optimization (PSO) was pioneered by Kennedy and Eberhart^64^ for optimization purposes. PSO, which is consisted of particles, iteratively searches for optimal value/goal. In the searching phase, the particles are responsible for adjusting their own positions in accordance with their own experiences and those of the other particles in the system. For the purpose of achieving the best position, each particle must follow the personal best position (PBEST) and global best position (GBEST). In the course of training process, each particle tends to move towards its own PBEST and GBEST, which is on the basis of a new velocity term and distance of its best positions in the learning phase. As a result, each particle’s new position is dependent upon the new velocity value in following iteration. Figure 2 depicts a PSO flowchart and how it works. More detailed explanations concerning PSO and its modeling procedure are available in literature^56,65^.

### Imperialist competitive algorithm (ICA)

**Figure 3:**
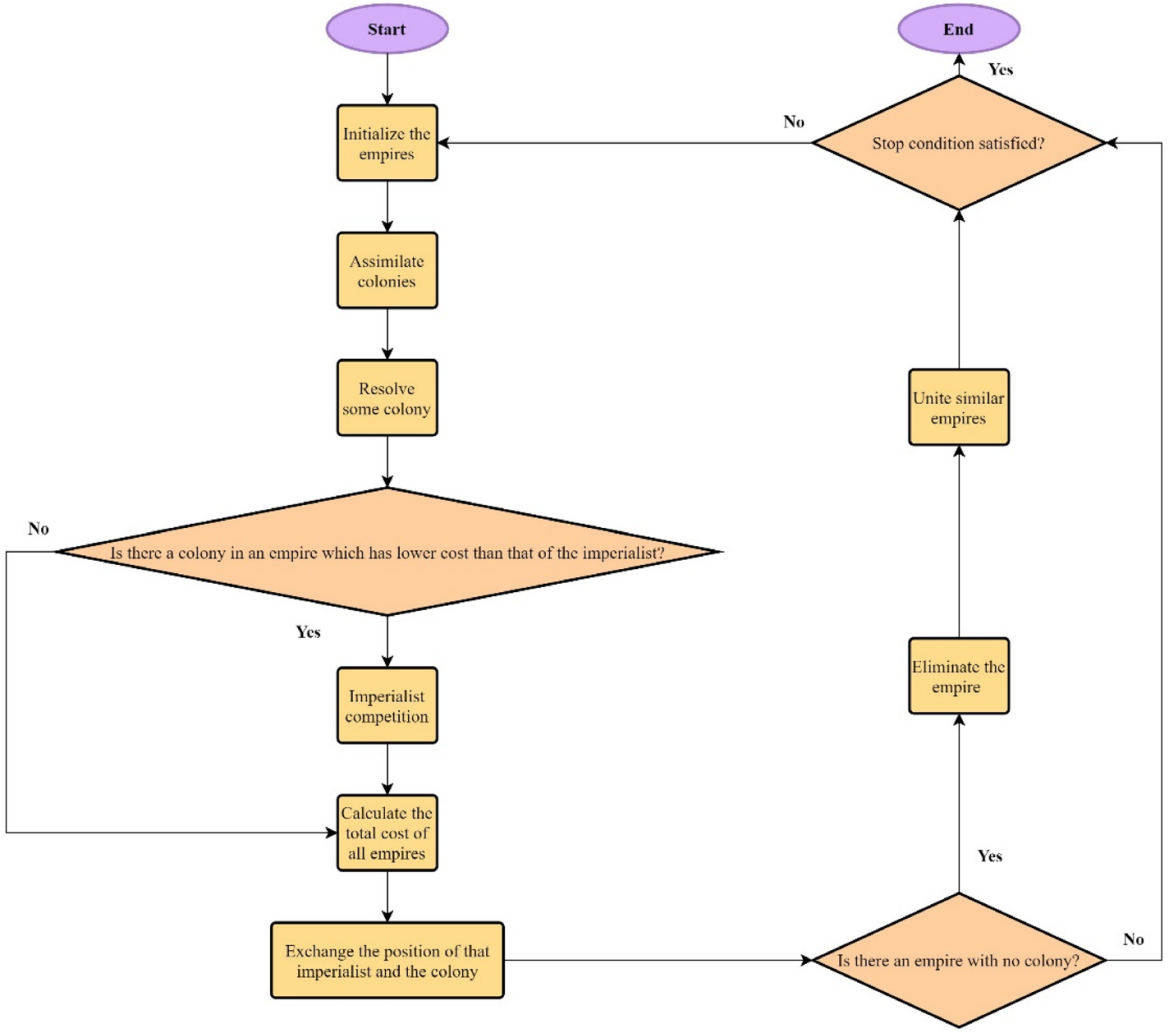
The flowchart of ICA algorithm structure.

Atashpaz-Gargari and Lucas^66^ developed the ICA technique in order to solve the optimization problems. ICA, which is known as a population-based algorithm for global search, begins its operation with a random initial population or candidate solution. In the scenarios performed by ICA, the countries of the highest power take the role of imperialist, whereas the rest of the countries are referred to as colony owned by imperialists. ICA is essentially designed on the basis of the real socio-politic competitions that normally take place amongst imperialists in the real world in order to take the possession of colonies. Each ICA involves three operators: assimilation, revolution, and competition. Assimilation leads the colonies towards becoming imperialist. The reason of such movement is the colonies’ eagerness for obtaining more power, higher cultural level, and stronger economy; an imperialist owns all of them. Revolution refers to the impulsive changes that may take place in the position of countries. During the assimilation and revolution operators, colonies get a chance to achieve position higher than their respective imperialist; in this condition, they may be able to take the empire’s control. On the other hand, the competition operator gives the opportunity to the imperialists to make every effort to possess more and more colonies. During this process, all empires use force to take the other empires’ colonies. All imperialists are potentially capable of possessing at least a single colony already possessed by the weakest empire; it completely depends on their power. This process is reiterated until only one empire remains in power and others empires are totally collapsed and shifted into colonies possessed by that most powerful one. Figure 3 illustrates flowchart of ICA process from start to end. For more information regarding ICA and its optimization process, other available studies can be considered and reviewed^66^.

### Genetic algorithm (GA)

**Figure 4:**
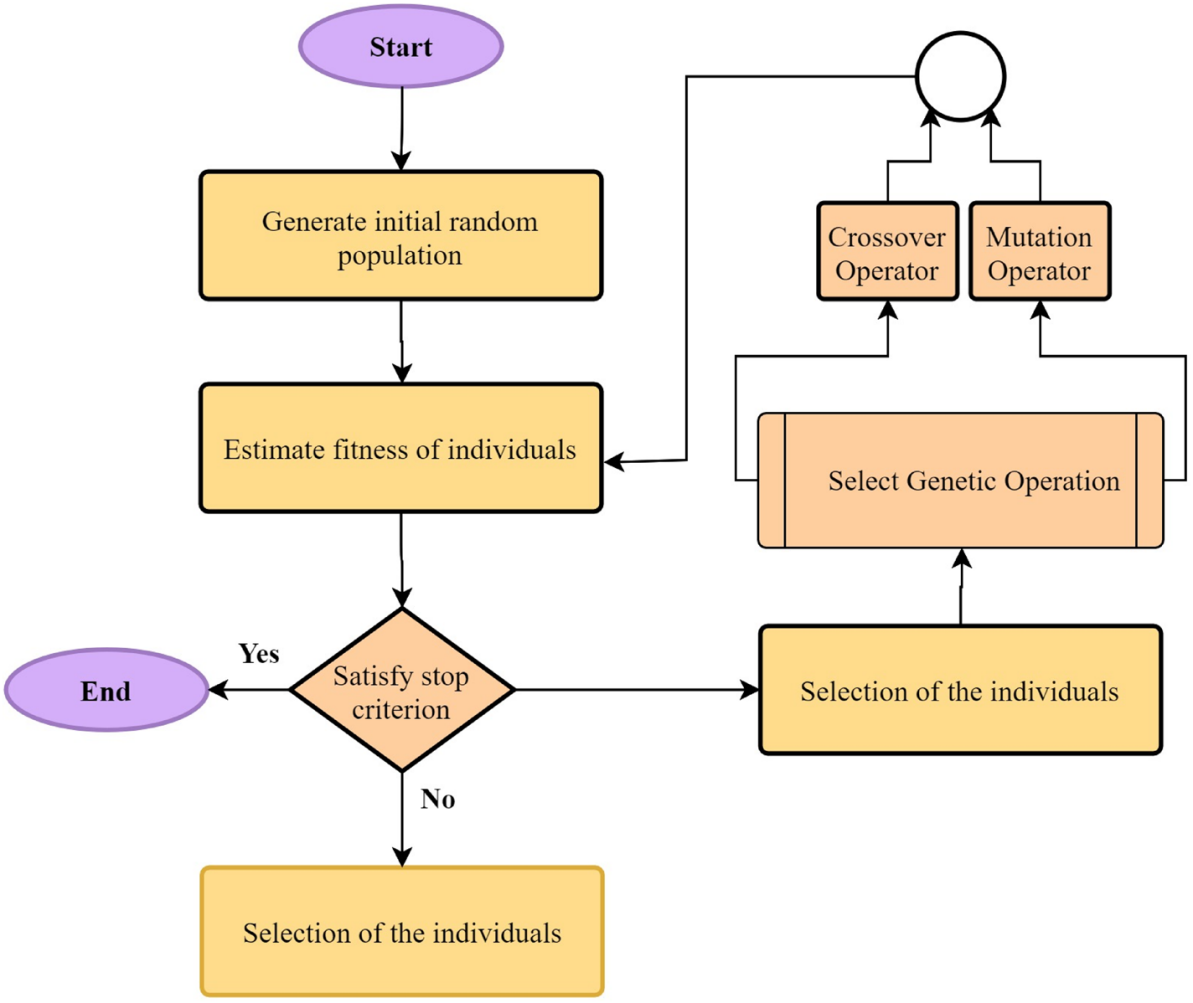
GA algorithm.

One of the most common algorithms that optimizes problems based on natural selection is the GA. This algorithm, originally introduced by Holland^67^, and it was modified several time by the other researchers^68^. The optimization of different problem conditions, such as static or dynamic, discrete and continuous problems, and a combination of them can be solved by the GA algorithm. Convergence and impact on the final results of various problems, due to the dependence on various variables such as coefficients and population, makes this algorithm highly sensitive. These conditions make it possible to use this algorithm with high precision^69^. The chromosomes used in the GA algorithm, with the same length, produce a new generation. In Fig. 4, a chromosome is shown, which is initially introduced as a measurable property and is selected randomly. Choosing the best generation is actually the end of a process that is identified by genetic operators. Here, Crossover and mutation operations are performed so that the final selection, which is actually the final and optimal answer, can be reached^56^.

### Artificial bee colony (ABC)

The ABC algorithm was developed by Karaboga^70^. His optimization algorithm was designed getting inspired by the bees’ social life. In this algorithm, each bee stands for a simple component. In case the bees create together a colony, then some complicated and coherent behaviors will appear, which are capable of forming an integrated system through which nectar of flowers can be discovered and exploited. Each colony comprises three colonies of bees, which perform a certain duty. The first group includes scouts whose responsibility is to explore new sources. The scouts do their searching task in a random way in the outlying environment. When they discover a source of food, they will store it in their memory. When each bee returns to the hive, it shares the information about the source of food with the other bees performing a waggle dance; then, it hires some other bees for the exploitation of the discovered sources. The second group of bees is consisted of the employed ones whose task is the exploitation of the predetermined sources of food. The third group comprises the onlookers which normally remain in the hive waiting for the other bees; then, following the exchange of information with other bees through the process of the waggle dance, they will select one resource considering the fitness of the answers for exploitation.

In general, ABC comprises four steps^71^: Stage 1: Initially, half of the bees population includes the employed bees and the remaining part includes the non-employed ones. Each source of food is allocated with an employed bee. In other words, in the scope of the answer, the number of sources of food shapes this algorithm’s initial solution. Then, each solution’s value needs to be computed by means of the relationship of the certain problem in hand.

Stage 2: A new answer is applied to each of the solutions considering the bellow relationship:

2 $$\begin{gathered} v_{i,j} = x_{i.j} + \varphi_{i.j} \left( {x_{i.j} - x_{k.j} } \right) \hfill \\ i \in \left\{ {1,2, \ldots BN} \right\} \hfill \\ j \in \left\{ {1, 2, \ldots P} \right\} \hfill \\ k \in \left\{ {1, 2, \ldots BN} \right\} \& k \ne i \hfill \\ \varphi \in \left[ { - 1.1} \right] \hfill \\ \end{gathered}$$

where $x_{i.j}$ stands for the parameter *j* from answer *i*, the $v_{i,j}$ represents the parameter *j* of the new answer, *i* denotes the number of one to the number of solution problems, *φ* signifies a random number within the interval [− 1,1], *k* shows a random number from one to the number of problem answers, *BN* represents the number of initial answers to the given problem, and *P* denotes the number of optimization parameters.

Stage 3: This step involves the calculation of the receiving bees probability from each of the sites using the equation bellow:

3 $$p_{i} = \frac{{fit_{i} }}{{\mathop \sum \nolimits_{n = 1}^{SN} fit_{n} }}$$

where $fit_{i}$ shows the source of the fitness of source *i*, and $p_{i}$ signifies the probability of the selection of source *i* by the onlooker bees. Several bees are allocated based on each item’s fitness. At this time, all of the available bees might be allocated to one food site depending on the fitness. When the value of each source is computed by means of Eq. (2); then, a new answer is produced for the answers chosen. If it gets a value higher than the former answer, the former answer will be replaced by the new one; otherwise, it will be fined.

**Figure 5:**
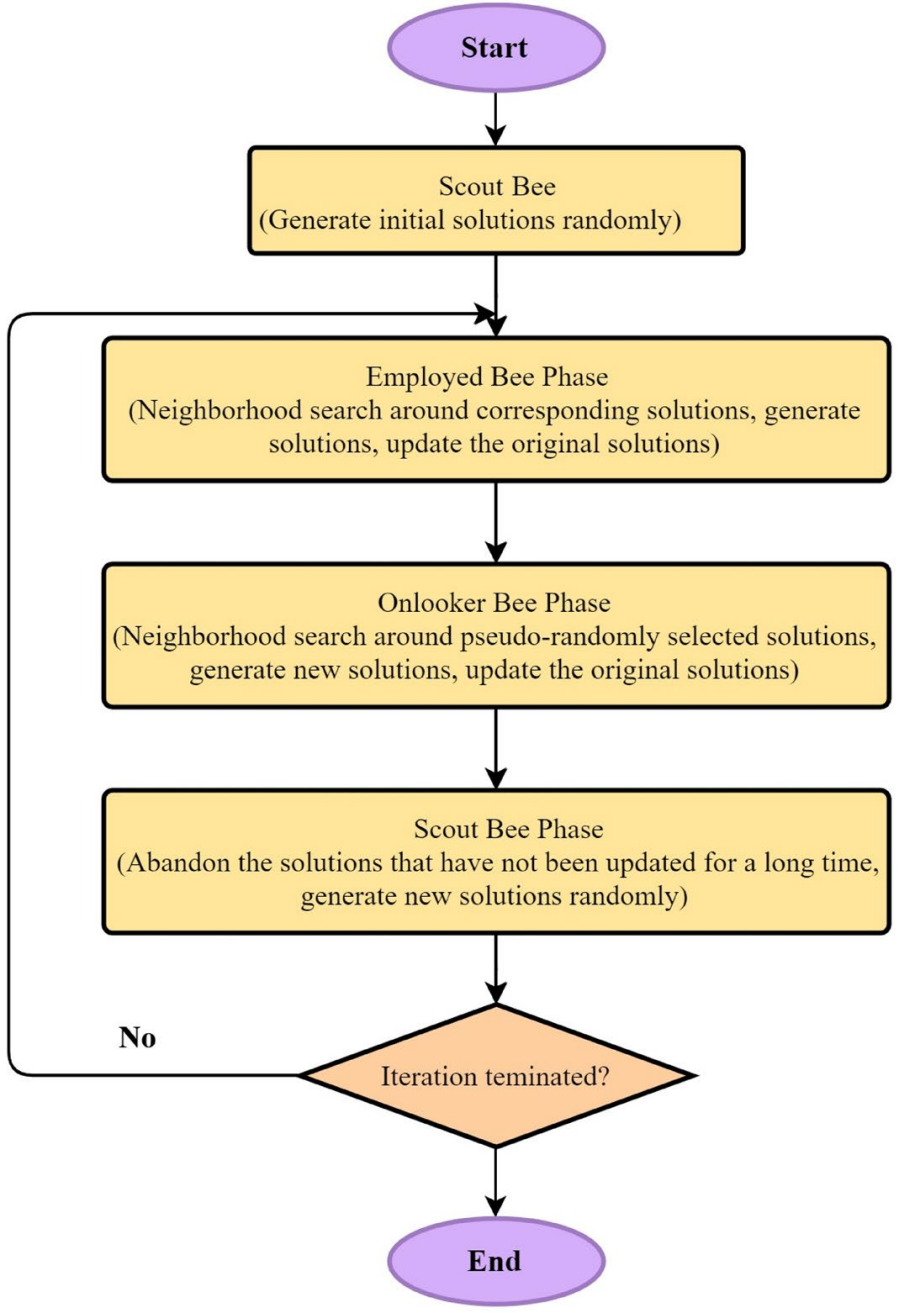
The flowchart of ABC algorithm.

Stage 4: If the counter of non-improved answer reaches the predefined limit (Cmax), the answer is replaced by a random answer. In this step, the termination conditions are also checked. If these conditions are met, the operation of the algorithm terminates; otherwise, it returns to the second step. The flowchart of ABC algorithm is presented in Fig. 5.

### Firefly algorithm (FA)

**Figure 6:**
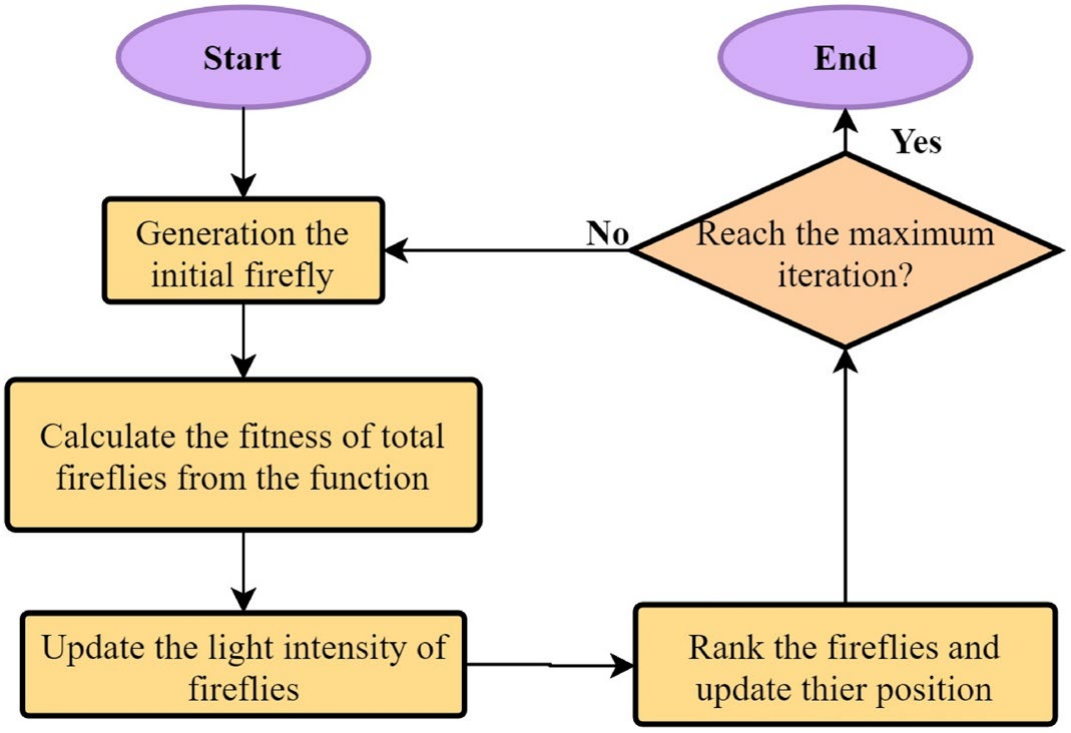
Flowchart of FA optimization algorithm.

The FA imitates the fireflies’ behaviors in their social settings. These insects conspicuously utilize their nature-gifted bioluminescence in order to release light in different flashing patterns. The fireflies’ flashing characteristics were found idealized by several researchers^72^ in order to develop a mathematical form of their behaviors. To be understood more easily, only three recognized rules of FA are presented as follows:

(1) The sex of all fireflies is the same; therefore, a firefly may be attracted to other fireflies irrespective of their sex.

(2) The brightness of a firefly is its key property to be attractive. In other words, when brightness of two fireflies is not at the same level, the brighter firefly attracts the insect of less brightness.

(3) A firefly’s brightness is related to the analytical shape of the cost function. A direct relationship is needed to be set between brightness and the cost function value so that the problem can be maximized. To be brief, Fig. 6 presents the pseudo code of the most important stages of the FA and the three rules corresponding to these steps.

The FA involves two significant issues: the light intensity variation and formulation of attractiveness. To make it simpler, the fireflies’ brightness is assumed to be applicable to the determination of their attractiveness level, which is in turn connected to the encoded objective function. On the other hand, attractiveness of the fireflies and light intensity are in an indirect relationship; attractiveness reduces with an increase of the distance from the source.

The following Gaussian form^72^ is applied to determine the influence of the inverse square law and absorption in most applications.

4 $$I\left( r \right) = I_{0} e^{{ - \gamma r^{2} }}$$

where the light absorption coefficient, $\gamma$, can be assumed as a constant value. A firefly’s attractiveness, $\beta$, can be measured using the following equation since the attractiveness of a firefly is directly related to the light intensity that is observable by the neighboring fireflies^72^.

5 $$\beta \left( r \right) = \beta_{0} e^{{ - \gamma r^{2} }}$$

where, $\beta_{0}$ shows the attractiveness at *r* = 0. A characteristic distance $\Gamma = \frac{1}{\surd \gamma }$ is defined by Eq. 5 where the attractiveness is varied considerably between $\beta_{0}$ and $\beta_{0} {\text{e}}^{{ - {\upgamma }r^{2} }}$.

The Cartesian distance $r_{ij} = \left\| {X_{i} - X_{j} } \right\|$ here defines the distance between any two fireflies *i* and *j* at *X*_*i*_ and *X*_*j*_, respectively.

6 $$\Delta X_{i} = \beta_{0} e^{{ - \gamma r^{2} }} \left( {X_{j}^{t} - X_{i}^{t} } \right) + \alpha \varepsilon_{i} , X_{i}^{t + 1} = X_{i}^{t} + \Delta X$$

The first equation here is connected to attraction, while the second equation is actually a randomization equation with *α*. $\varepsilon_{i}$ signifies a vector of the random numbers that are attained from a Gaussian distribution. The value of the step size is randomly achieved as follows:

7 $$L\left( s \right) = A_{s}^{ - 1 - q} , A = q\Gamma \left( q \right)\sin \left( {\frac{\pi q}{2}} \right)/\pi$$

where, $\Gamma \left( q \right)$ signifies a Gamma function and *q* denotes the distribution exponent. Based on the implementation viewpoint, the last phrase should be replaced by the phrase of $\alpha L\left( s \right)$ to produce the solution by means of Eq. (6). In case of most of the problems, a fixed value of $\alpha$ = 0.01 is applicable, whereas for all of the simulations, the phrase of *q* = 1.5 can be employed. An advantage of the FA in doing their activities are not dependent on each other, hence being of a high applicability to parallel implementations. Fireflies normally make a closer aggregation around each optimum; as a result, this algorithm can have a better performance in comparison with PSO and GA.

### Hybrid ANN-based models

Numerous scholars working in the context of engineering science^57,73,74^ have attempted to improve the competencies of the ANN models by means of OAs, e.g., PSO, GA, ICA, ABC, and FA. BP is normally weak in exploring the global minimum accurately; therefore, it is possible for ANN to attain an undesirable result^75^. Although ANN has a higher probability of being caught in local minima, the optimization algorithms are capable of solving this problem through setting the ANN’s weights and biases. In this condition, the search space may be faced with global minimum because of using OAs. In this case, ANN is determinant of the most efficient results obtained by the hybrid ANN-based models such as PSO-ANN, GA-ANN, ICA-ANN, ABC-ANN and FA-ANN. In this study, five hybrid models, namely PSO-ANN, GA-ANN, ICA-ANN, ABC-ANN and FA-ANN are selected and developed to predict PPV induced by blasting. Then, the prediction performance of these models is compared to choose the best hybrid ANN-based model.

## Case study and input parameters

### Study area and data collection

**Figure 7:**
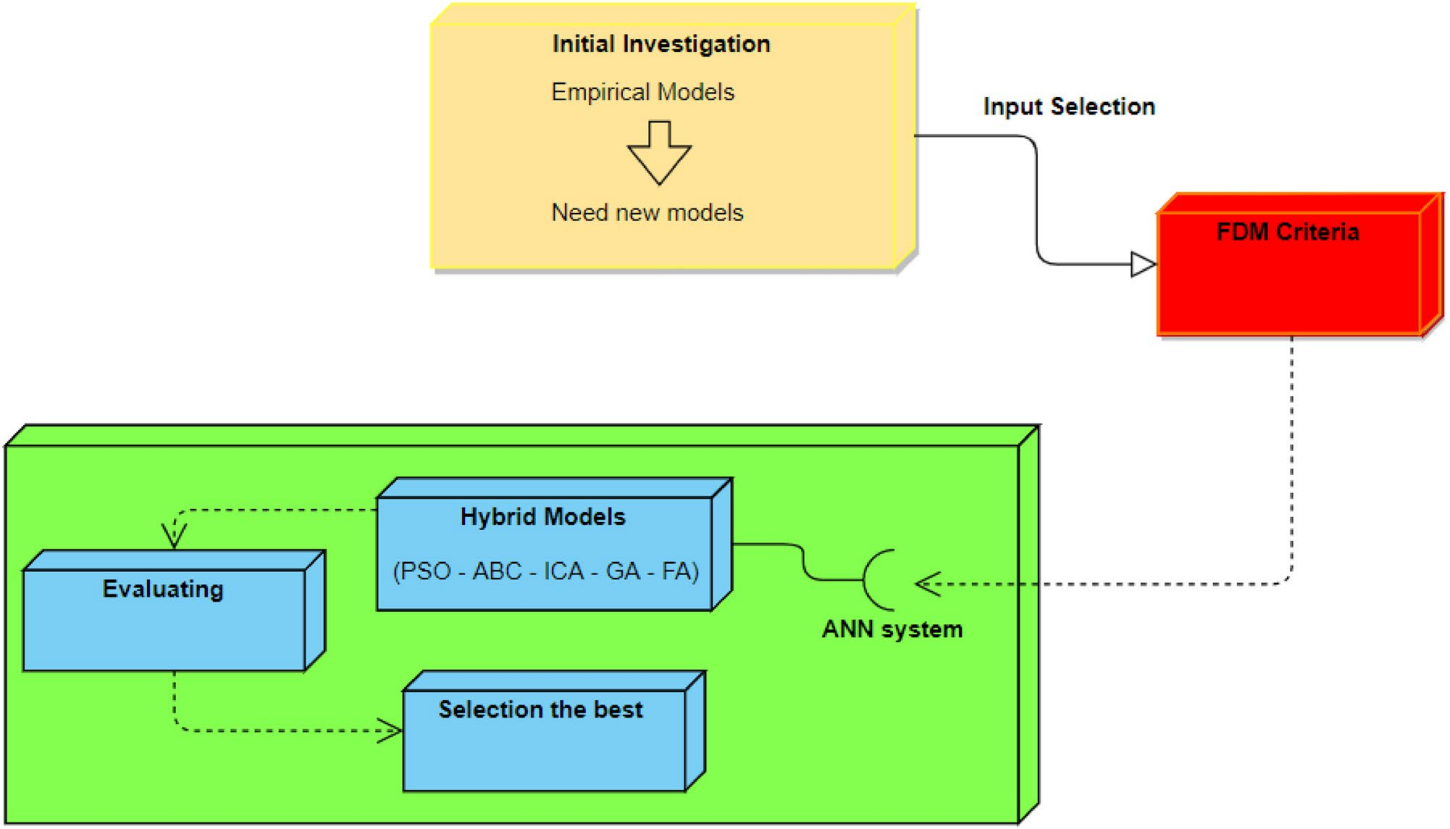
The general flowchart of this study.

The present research was carried out at the quarry site of Hulu Langat which is located in the south of Selangor state, Malaysia. More specifically, this is at a latitude of 3′ 7′ 0″ N and a longitude of 101′ 49′ 1″ E. The quarry consists of granitic rock and it is capable of producing large amounts of aggregate (more than 300,000 tons per month). In general, the blasting operations are typically carried out by means of blast holes of 89 mm in diameter. For the explosive material, ammonium nitrate and fuel oil (ANFO) was used and for initiating the blast, dynamite was employed at predefined spots. Gravels were used in order to stem required blast holes. A total of 88 datasets were gathered during 6 months. In the course of gathering required data, some important blasting parameters were attained, e.g., the number of holes, depth of the holes, length of stemming, maximum charge per delay, spacing, burden, sub-drilling, and powder factor. The VibraZEB seismograph was applied in each blast in order to record the values of PPV. Recording of all values related to PPV was done in front of the quarry bench and almost vertical to that. The location of the workshops and crushing plant was around 400 m of the face of quarry, whereas the closest inhabited region was roughly 800 m to the west of that. As a result, the values set to be the distance from the monitoring point to the free face were 300, 600, and 700 m. The data collected from the study area together with their unit and some statistical information are shown in Table 1. The average values of 14.4 m, 84.5 kg, 0.8, 2.7 m, 35.3 cm, 0.9 kg/m^3^, 59.4%, 553.3 m, 38.1 and 3.5 mm/s were obtained for HD, MC, BS, St, SD, PF, RQD, D, No. H and PPV, respectively. The general flowchart of this study is presented in Fig. 7. According to this flowchart, after initial evaluation of the data and proposing empirical model, FDM is used to select input parameters from all data. Then, 5 hybrid ANN-based models are constructed to predict PPV resulting from blasting. After constructing the hybrid models, they are evaluated using performance indices and the best one among them is selected.

**Table 1:** The data collected from the study area.

Parameter/Unit	Symbol	Range
Powder factor/kg/m^3^	PF	0.4–1.18
Stemming length/m	St	1.9–3.6
Rock quality designation/%	RQD	41–77
Distance from the free face/m	D	300–700
Hole depth/m	HD	10–17
Burden to spacing/–	BS	0.7–0.92
Number of hole/–	No. H	15–63
Charge per delay/kg	MC	56.3–101.6
Sub-drilling/cm	SD	25–45
Peak particle velocity/mm/s	PPV	1.1–9.5

### Input selection by FDM

This section presents the process of input selection by FDM to estimate PPV. It is important to mention that the same data has been published in another study of PPV prediction by Hajihassani et al.^76^. In the published paper, the authors used HD, MC, BS, St, SD, PF, RQD, D, and No. H to predict PPV developing a hybrid intelligent model. Hajihassani et al.^76^ used 9 parameters as model inputs to predict PPV and as a fact, preparation of these inputs is difficult in practice. Therefore, the present study is aimed to decrease the number of inputs in the way to obtain the similar prediction performance. On the other hand, Jahed Armaghani et al.^77^ mentioned that a prediction technique with minimum number of model inputs is of interest and attention as it is able to decrease the complexity of the developed model. Hence, to determine the most significant variables affecting the results of PPV, FDM was used. FDM is an expert-based approach which is a powerful tool to acquire the knowledge of experts in terms of the importance of variables. First, the affecting variables were investigated through reviewing the literature and then, Malaysian experts were asked to give their opinions regarding them. Subsequently, based on the results of FDM, the most significant variables were determined.

According to Fig. 7 (research framework), the initial step for developing PPV predictive models is to determine its most significant variables. To do that, Delphi method was used to acquire the knowledge of experts in current research area. Delphi technique was developed in 1963, since then modified by many researchers. The modified method consist of FDM, fuzzy with Delphi, enhanced FDM which have been modified and used by Ishikawa et al.^78^, Yih^79^, and Mahdiyar et al.^80^, respectively. The advantages and drawbacks of these methods are discussed in the study conducted by Mahdiyar et al.^80^. To determine the significance of the variables, five stepes were carried out as follows:

**Step 1.** Investigating the variables affecting PPV results through extensive reviewing of the literature.

**Step 2.** Distributing the questionnaire to the experts. The experts were asked to determine the importance of each variable using five-scale questionnaire, “very low,” “low,” “medium,” “high,” and “very high.” Table 2 shows the linguistic scales for the variables in this study.

**Table 2:** Linguistic scales relative used in this study.

Linguistic variables	Scales
Very low	[(0.1, 0.1); 0.1; (0.2, 0.25)]
Low	[(0.15, 0.2); 0.3; (0.4, 0.45)]
Medium	[(0.35, 0.4); 0.5; (0.6, 0.65)]
High	[(0.55, 0.6); 0.7; (0.8, 0.85)]
Very high	[(0.75, 0.8); 0.9; (0.9, 0.90)]

**Step 3.** Collecting the experts’ feedback and calculating their consensus value. Assume having *i* of experts and *j* variables, the experts’ opinions are calculated as follows:

$$\begin{gathered} \tilde{A}_{{inv_{j} }} = \left[ {\left( {l_{2} , l_{1} } \right)_{j} , m_{2j} ,\left( {u_{1} , u_{2} } \right)_{j} } \right]\,\,\,\, i = \, 1, \, 2, \, 3, \ldots ,n, \, and\,\,\,j = \, 1, \, 2, \, 3, \ldots ,m. \hfill \\ \left( {l_{2} , l_{1} } \right)_{j} = \min \left\{ {\left( {l_{2} , l_{1} } \right)_{ij} } \right\} \hfill \\ m_{2j} = \frac{1}{n}\mathop \sum \limits_{i = 1}^{n} m_{2ij} \hfill \\ \left( {u_{1} , u_{1} } \right)_{j} = \max \left\{ {\left( {u_{1} , u_{1} } \right)_{ij} } \right\} \hfill \\ \end{gathered}$$

where $\tilde{A}_{{inv_{j} }}$ is interval-valued fuzzy weighting of No. *j* variable, $\left( {l_{2} , l_{1} } \right)_{j}$ and $\left( {u_{1} , u_{1} } \right)_{j}$ are the bottom and top experts’ appraisal value, respectively. $m_{2j}$ is the mean of all the experts’ appraisal.

**Step 4.** Defuzzifying the fuzzy weight of each variable using following equation:

8 $$S_{j} = \frac{{l_{2j} + l_{1j} + m_{2j} + u_{1j} + u_{2j} }}{5},\quad {\text{for}}\,\,j = {1},{2}, \ldots ,m$$

**Step 5.** Determine the significant variables. A threshold value was defined as the mean of all defuzzified values. Then, the variables with defuzzified value of less than threshold were not considered as significant factor.

**Figure 8:**
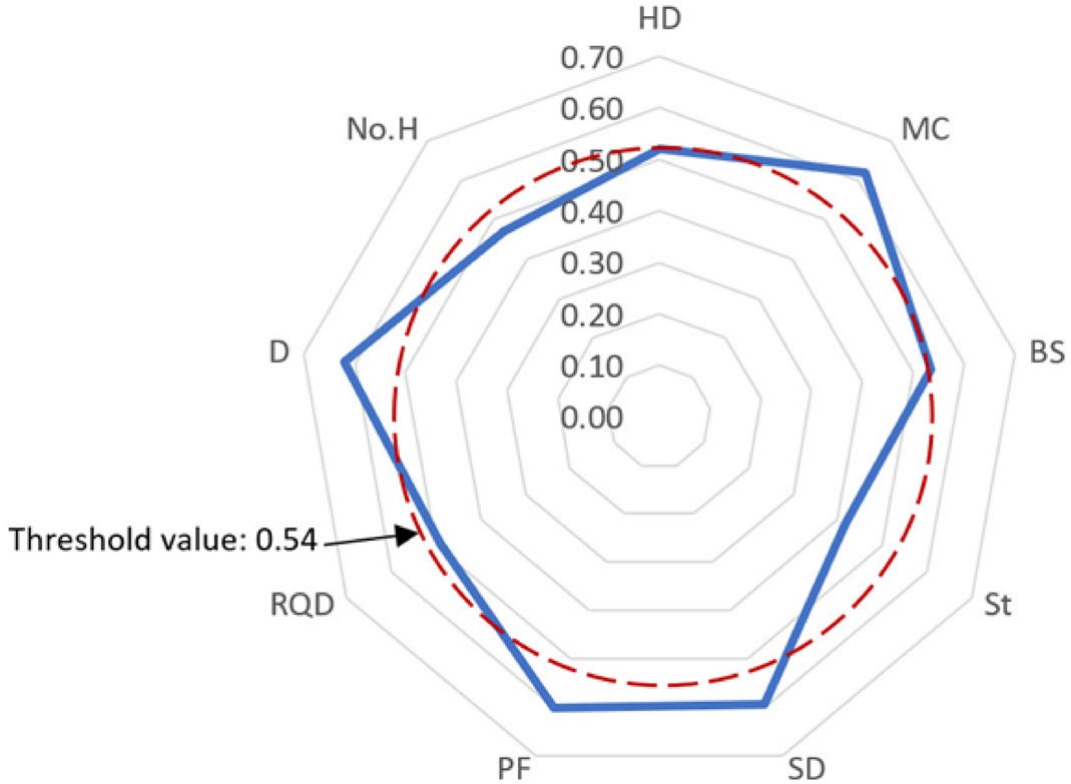
Variables’ defuzzified weights and the threshold value.

As mentioned before, the experts were selected from Malaysian professionals those have vast experience in field of blasting. The online questionnaire was prepared and distributed among the experts according to the collected data of this study and analyzed. Table 3 shows the results of the analysis. The higher the value of defuzzified weight, the more important the variable. As a result, *MC*. *D*, *PF*, *SD*, *BS*, *HD*, *RQD*, *No. H*, and *St* are the most significant variables affecting PPV results in a descent order. The threshold value was calculated based on the average value of all variables’ defuzzified values. As it can be seen in Fig. 8, four parameters (*MC*, *D*, *PF*, and *SD*) out of nine variables are out of the threshold boundary and then perceived as significant variables. The other five variables are decided to be rejected for the next step of analysis in this research. It is worth mentioning that *MC* and *D* are the most important parameters in well-established previous empirical equations^9,13,15^. Based on results of this section, *MC*, *D*, *PF* and *SD* were selected as the most influential parameters on PPV and they were used as model inputs in modeling of PPV in this research.

**Table 3:** The results obtained from FDM technique.

Variable	Defuzzified weight based on all expert’s feedbacks	Decision
HD	0.52	Reject
MC	0.62	Select
BS	0.53	Reject
St	0.42	Reject
SD	0.59	Select
PF	0.60	Select
RQD	0.49	Reject
D	0.62	Select
No. H	0.47	Reject
Threshold	0.54

## Proposing empirical equation

As mentioned earlier, many researcher tried to develop empirical formulas for prediction of PPV. Their formulas have been developed based on 2 parameters; maximum charge per delay (kg) and distance from free face of blast (m). According to the most popular empirical PPV formula proposed by Duvall and Petkof^10^, prediction of PPV should be done through the use of scaled distance (SD) which is presented as follows:

9 $$SD = \left( {\frac{D}{{\sqrt {MC} }}} \right)$$

where, *MC* and *D* are the maximum charge per delay (kg) and the distance from the free face (m), respectively. Then, the values of PPV can be estimated using the following equation type^10^:

10 $$PPV = K \left( {SD} \right)^{B}$$

where, *B* and *K* are site constants.

In this study, through the calculation of SD values, a PPV formula has been developed empirically as follows:

11 $$PPV = 638.41 \left( {SD} \right)^{ - 1.346}$$

**Figure 9:**
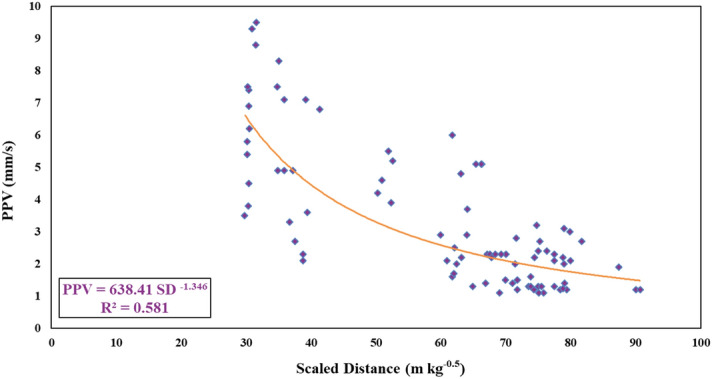
The relationship between SD values and measured PPV values.

The coefficient of determination (R^2^) value equal to 0.581 for the proposed PPV formula shows that the obtained results are suitable and meaningful. Figure 9 displays the relationship between SD values and measured PPV values and also the proposed PPV formula. In order to show ability of this formula, the most popular published PPV formulas were considered and applied on the data of this study (Table 4). The PPV values were obtained for all equations presented in Table 4 using 88 datsets of this study and their R^2^ results are tabulated in Table 5. As it can be seen, R^2^ values of 0.124, 0.125, 0.124, 0.127, 0.121, 0.125, and 0.581, were obtained for PPV equations of Duvall and Petkof, Langefors–Kihlstrom, Davies et al., Indian Standard, Ghosh–Pal Roy, and this study, respectively. The results show that the proposed formula in this study is able to predict PPV values better than the most popular published PPV equations. Therefore, it can be concluded that the equation proposed in this study can predict with suitable level of accuracy, however, in order to get higher performance capacity for PPV estimation, there is a need to apply and develop new computational techniques using not only 2 input parameters but also more than that (*MC*, *D*, *PF* and *SD*). Hence, in this study, 5 hybrid ANN-based models are used and developed to predict PPV induced by blasting.

**Table 4:** The most popular previously proposed PPV equations.

Equation	Equation	Site constant for granite
USBM by Duvall and Petkof^10^	$PPV = K\left[ {D/\surd MC} \right]^{ - B}$	*K*: 179.31, *B*: 1.09
Langefors–Kihlstrom^81^	$PPV = K\left[ {\surd \left( {MC/D^{2/3} } \right)} \right]^{B}$	*K*: 44.43, *B*: − 1.18
General predictor by Davies et al.^82^	$PPV = KD^{ - B} (MC)^{A}$	*K*: 212.27, *B*: 1.09, *A*: 0.52
Bureau of Indian Standard^13^	$PPV = K\left[ {\left( {MC/D^{2/3} } \right)} \right]^{B}$	*K*: 6.33, *B*: 0.22
Ghosh–Daemen predictor^83^	$PPV = K\left[ {D/\surd MC} \right]^{ - B} e^{ - \alpha R}$	*K*: 780.36, *B*: 1.26, *α*: 0.0004
CMRI by Pal Roy^15^	$PPV = n + K\left[ {D/\surd MC} \right]^{ - 1}$	*K*: 168.91, *n*: 1.57

*K, B, A, α* and *n* are site constants.

**Table 5:** Comparison of R^2^ results in predicting PPV.

Empirical PPV equation	R^2^
USBM by Duvall and Petkof^10^	0.124
Langefors–Kihlstrom^81^	0.125
General predictor by Davies et al.^82^	0.124
Bureau of Indian Standard^13^	0.127
Ghosh–Daemen predictor^83^	0.121
CMRI by Pal Roy^15^	*0.125*
This study	0.581

## Developing hybrid ANN-based models

The present section describes the implementation process of the hybrid ANN models, i.e., ICA-ANN, PSO-ANN, ABC-ANN, FA-ANN and GA-ANN for the prediction of PPV induced by blasting. In fact, the effects of ICA, GA, ABC, FA and PSO on optimizing weights and biases of ANN are investigated for prediction of PPV. We attempted to find out the parameters that can affect ICA, GA, ABC, FA and PSO, so that we can achieve a higher level of accurateness in terms of predicting PPV.

### ICA-ANN

**Figure 10:**
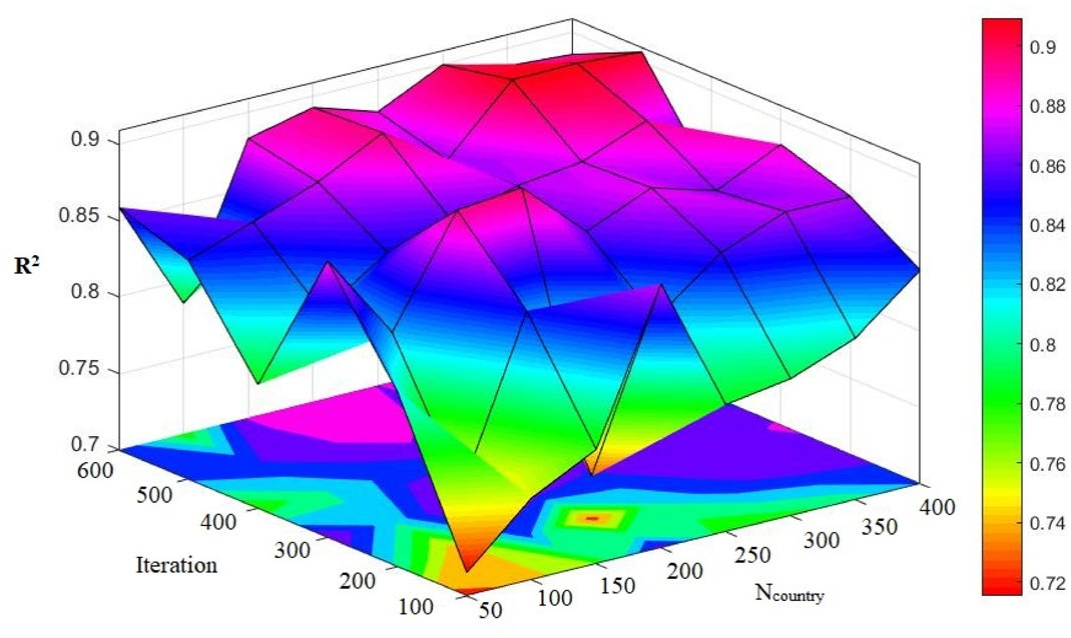
Simulation results of the impact of two N_country_ and iteration parameters on the developed models.

It is clear that for the achievement of an ICA-ANN model with the highest quality of performance, we need to examine the key parameters that have impact on the ICA. Therefore, it is necessary to determine the architecture of ANN before starting any experiment on the ICA parameters. It was done through a trial-and-error process and the architecture of 4 × 8 × 1 (or a model with 8 hidden nodes) obtained superior results, hence being applied to all hybrid models considered in this research. The key parameters affecting on ICA, as noted before, are the three parameters i.e., number of decade (N_decade_), number of country (N_country_), and number of imperialism (N_imp_). For the determination of a proper value of the N_imp_ parameter, numerous models were constructed setting values ranging from 5 to 40, with incremental step 5 and N_decade_ and N_country_ were fixed at 100 and 400, respectively. The obtained results confirmed that when N_imp_ was set to 5, the model could have its highest efficiency. In another experiment aiming at finding the most appropriate value for the N_decade_ parameter (see Fig. 10), a number of models with different N_decade_ values i.e., 50, 100, 150, 200, 250, 300, 350, and 400 were formed; then the models were evaluated considering their root mean square error (RMSE). The RMSE results were not changed after the N_decade_ = 500. In the final step, we set N_imp_ to 5 and N_decade_ to 500; then several ICA-ANN models were constructed and their efficiencies based on R^2^ and RMSE performance indices (PIs) were calculated (see Table 6).

**Table 6:**
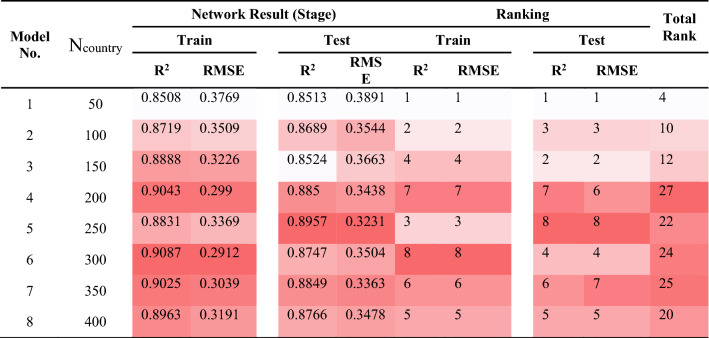
The results of ICA-ANN for estimating PPV.

For the selection of the hybrid models with the highest quality performance, the ranking method proposed by Zorlu et al*.*^84^ was employed. In their study, a comprehensive version of this method was accessible, according to an allocated rank to each PI result. For instance, amounts of 0.8508, 0.8719, 0.8888, 0.9043, 0.8831, 0.9087, 0.9025 and 0.8963 were obtained for R^2^ of train stage of model numbers 1 to 8, respectively. Then, amounts of 1, 2, 4, 7, 3, 8, 6, and 5 were assigned for their rankings, respectively. In case of the RMSE results, the same process was done. According to the results, model 4 (*Nc*_*ountry*_ = 200) with value of 27 (i.e., a total rank), was selected. In next sections, we will evaluate the results obtained from the selected ICA-ANN.

Here, each row of the models is colored exclusively; the higher intensity of the red color, the higher score it gets in comparison with the other scores within that column. On the contrary, when the intensity of the color lowers (lighter color), the parameter is assumed lower than the other parameters of the column. As a result, for training set, the model No. 4 provides a high intensity of red color in the R^2^ column (see Table 6). This way, in each of the columns, the best items are determined, while in the last column, an item that is shown collectively better (regarding the red color intensity) is chosen. Accordingly, the best models are selected by means of the new method. In this process, we made use of the coding technique for the determination of the colors intensity level.

### PSO-ANN

**Figure 11:**
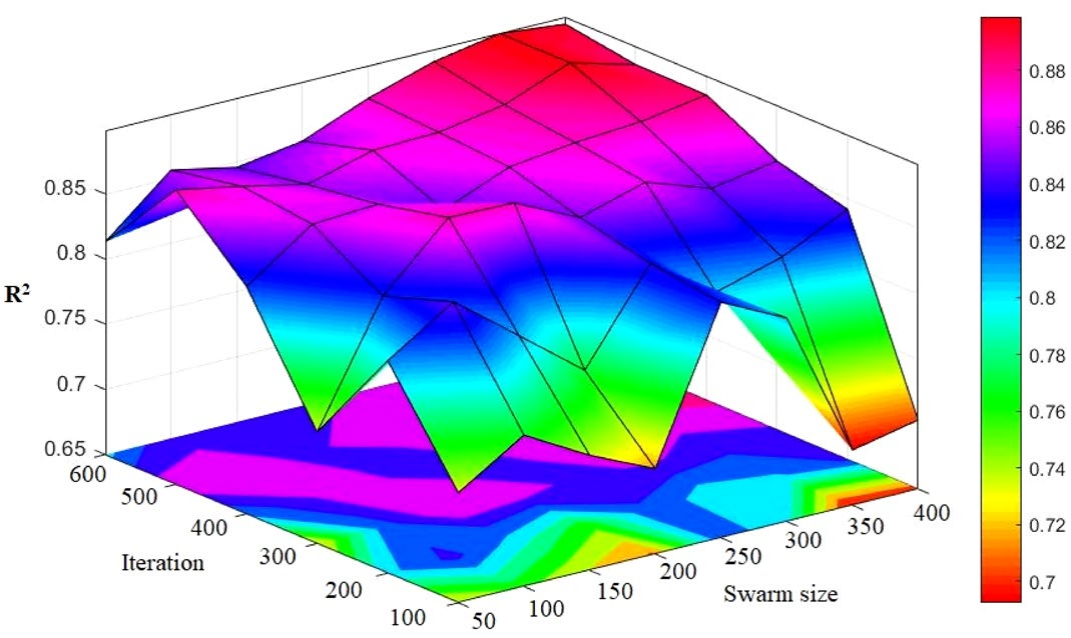
Simulation results of the impact of two swarm sizes and iteration parameters on the developed models.

As explained earlier, the PSO algorithm is significantly under the influence of a number of parameters such as inertia weight. Literature consists of numerous studies^85,86^ indicating that when the inertia weight is set at 0.25 and the coefficients of velocity equations are fixed at 2, desirable results can be obtained in other implemented PSO works. As a result, in all of the PSO-ANN models, the same values were applied. For the purpose of determining the most appropriate value of the number of iterations (see Fig. 11), different models configured using various amounts of swarm size in a range of 50 to 400, with incremental step 50. The models performances were examined considering their RMSE. Therefore, any change was not subjected to the RMSE results after the swarm size of 500. To determine the most proper value for the swarm size parameter, totally eight PSO-ANN models were configured for the prediction of PPV (see Table 7). Parallel to the former section, the simple ranking system was applied in this stage. According to the total rank values listed in Table 7, model No. 7 demonstrated the best results of the system. In this model, R^2^ of 0.8995 and 0.8749 were acquired in case of train and test data samples, respectively. In next sections, we will discuss the evaluations carried out on the selected PSO-ANN model.

**Table 7:**
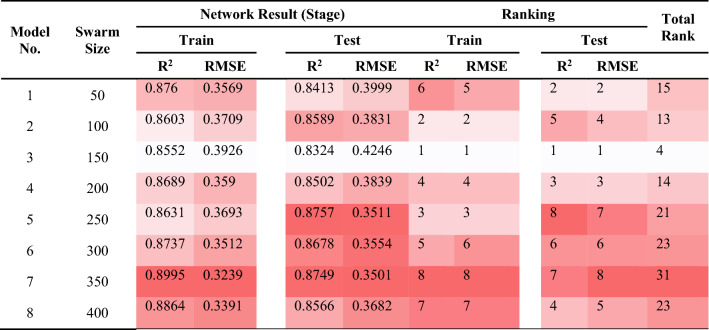
The results of PSO-ANN for estimating PPV.

### GA-ANN

**Figure 12:**
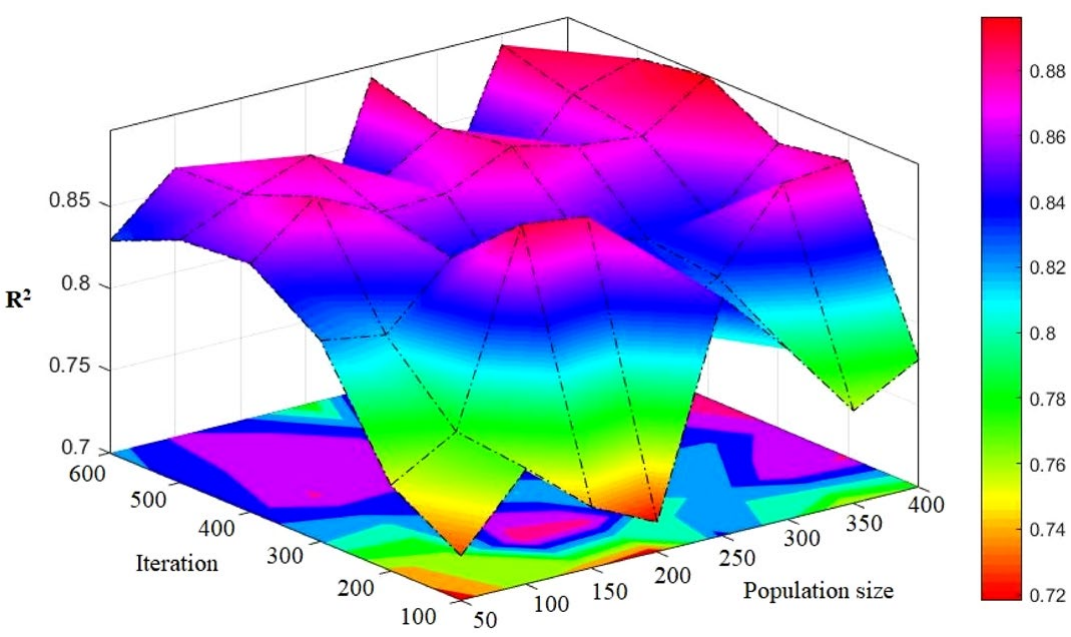
Simulation results of the impact of two population sizes and iteration parameters on the developed models.

GA, as noted before, affects the performance of ANN through optimizing its weights and biases. For the formation of a hybrid GA-ANN model, first, those parameters of GA that have the highest influence need to be identified. The recombination percentage and mutation probability values were fixed at 9% and 25%, respectively. A single point possibility of 70% was used as a cross-over operation. After that, it is turn to determine and make use of the maximum number of generation (G_max_). A parametric research was carried out in order to examine the impacts of G_max_ upon the performance of the network. In an attempt made to optimize the number of generations, as depicted in Fig. 12, we defined the value of 600 generation as the termination criteria, and the attained values of RMSE were considered. When the number of generations was fixed at 500, no change occurred to performance of the network; thus, this value was set as the optimum number of generations in the design process of the GA-ANN models. The final step involved the formation of several hybrid GA-ANN models (see Table 8) aiming at the determination of the most appropriate size of the population, which could be selected from a range of values from 50 to 400, with incremental step 50. The obtained results confirmed that if the value of population size is set to 250 with total rank of 27, a higher quality performance can be achieved regarding the R^2^ and RMSE indices. Later, a detailed explanation will be presented in case of the model number 5 (i.e., the selected model).

**Table 8:**
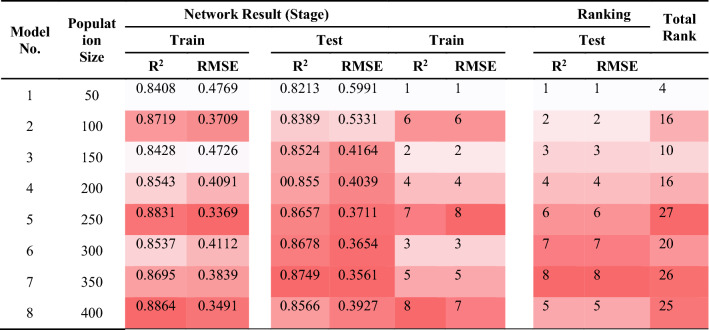
The results of GA-ANN for estimating PPV.

### ABC-ANN

**Figure 13:**
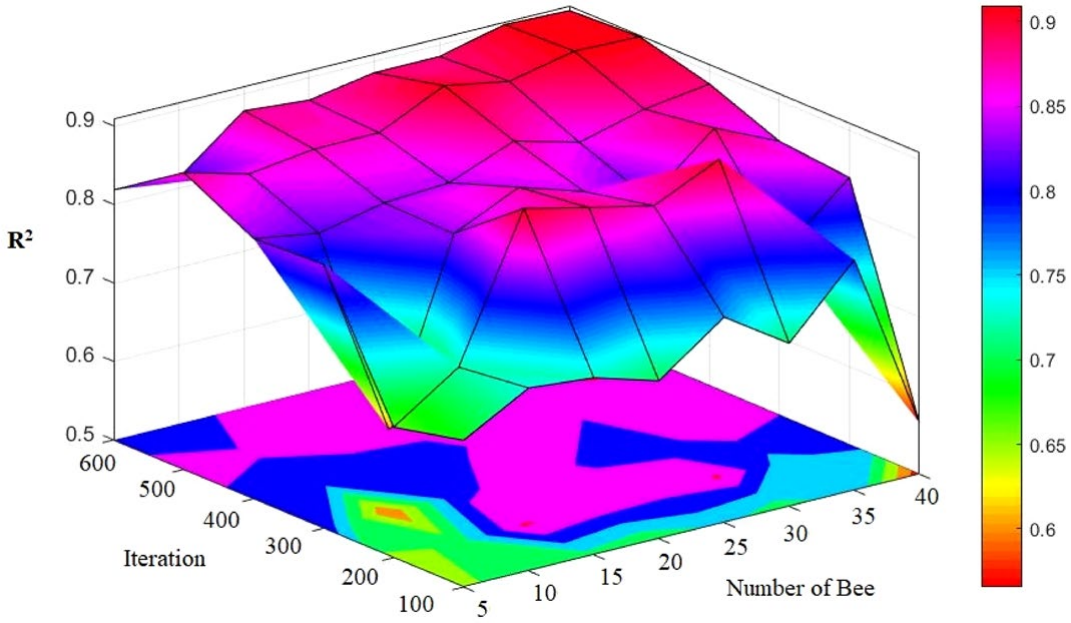
Simulation results of the impact of two Number of Bee and iteration parameters on the developed models.

The ANN’s performance quality was attempted to be enhanced with the help of the ABC algorithm. In this part of the study, ABC is used aiming at the exploration of the appropriate weights and optimizing them. It is important to mention that an increase in the bees No. lead to rising their capability of detecting additional ranges. Like the previous sections, the ABC-ANN models were constructed to predict PPV values. As can be seen in Fig. 13, 50–400 bees were distributed in the network in order to examine the effects of the bees on the performance of the model. It was expected to get better results with rising the number of bees; though, after 600 iteration, approximately all answers approach together. This is due to the fact that bees normally come together around the spots wherein the best answer is laid. This is why ABC enjoys a higher speed compared to other algorithms; in addition, the answer explored is typically of a higher performance quality. The values that were attained from the ABC-ANN models to predict PPV are presented in Table 9. Based on the ranking method, the model No. 7 that contained totally 35 bees was selected as the most suitable one in regard to the total R^2^ and RMSE scores. The values of R^2^ and RMSE for train and test stages of model No.7 were 0.9095, 0.2839, 0.9094 and 0.2905, respectively. The best ABC-ANN model will be discussed with more details later.

**Table 9:**
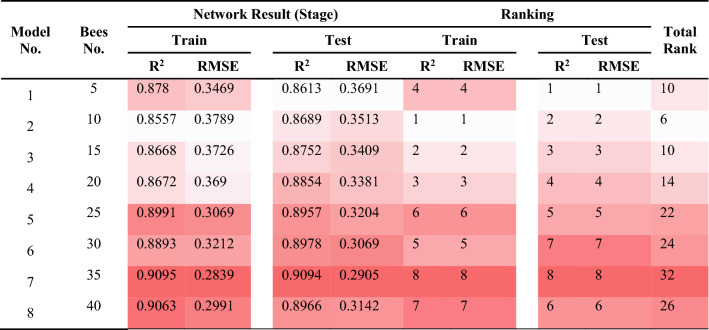
The results of ABC-ANN for estimating PPV.

### FA-ANN

**Figure 14:**
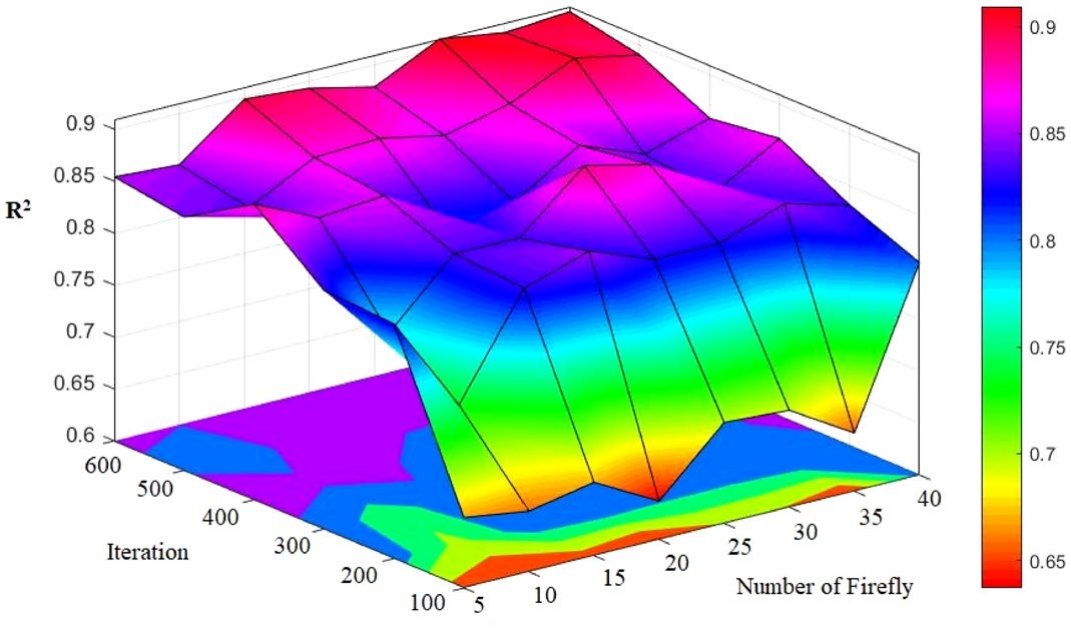
Simulation results of the impact of two number of firefly and iteration parameters on the developed models.

When working with FA, users need to determine a number of internal parameters significantly affecting the convergence behaviors at various degrees. A variety of ranges needed to be taken into account in the production process of the initial solution so that we can make it certain that the optimized solution does not have any sensitivity to it. This study adopted two approaches for the achievement of this objective: (a) the uniform distribution of the initial solutions sampling in a way to avoid biasing any region of search space; (b) production of each firefly in a way that it can have the farthest distance with the other fireflies so that they can discover the search space more effectively. To each initial population, the optimization process was applied and it was iterated for 100 times. The conclusion was that the initial guess considerably affects the optimization result, and the results were established through statistical measures, e.g., the corresponding standard deviation and the mean value of cost function. This approach was shown more efficient compared to those method depending only upon a number of optimization. To obtain the results of a higher precision, rigorous sensitivity analysis was uses in the examination of the impacts of two parameters, attractiveness and population. The analysis confirmed that if the population size is set to a value ranging between 5 and 50, we can achieve desirable results in case of common applications; however, in case of the problems with a higher complexity level, it needs to be risen to some extent. A total of 50 fireflies is sufficient in virtually all problems; greater numbers may lead to a significant rise the computation time. In the different FA-ANN models considered in the present study, 600 iteration and various numbers of fireflies (in the range of 5–40) were taken into account (Fig. 14). When the number of fireflies was set to 30 with minimum differences, the most appropriate pattern was obtained and used. In this condition, the optimum number of bees was also applied to the minimization of the computation time of analysis. The best model is model No. 6 with values R^2^ = 0.9133 and 0.9097 for training and testing respectively (see Table 10). This selected FA-ANN model will be described with more details in the next section.

**Table 10:**
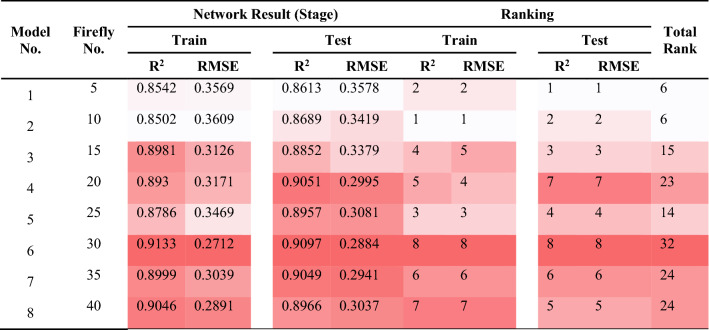
The results of FA-ANN for estimating PPV.

### Ethical approval

The authors confirm that all methods were carried out in accordance with relevant guidelines and regulations.

## Results and discussion

**Figure 15:**
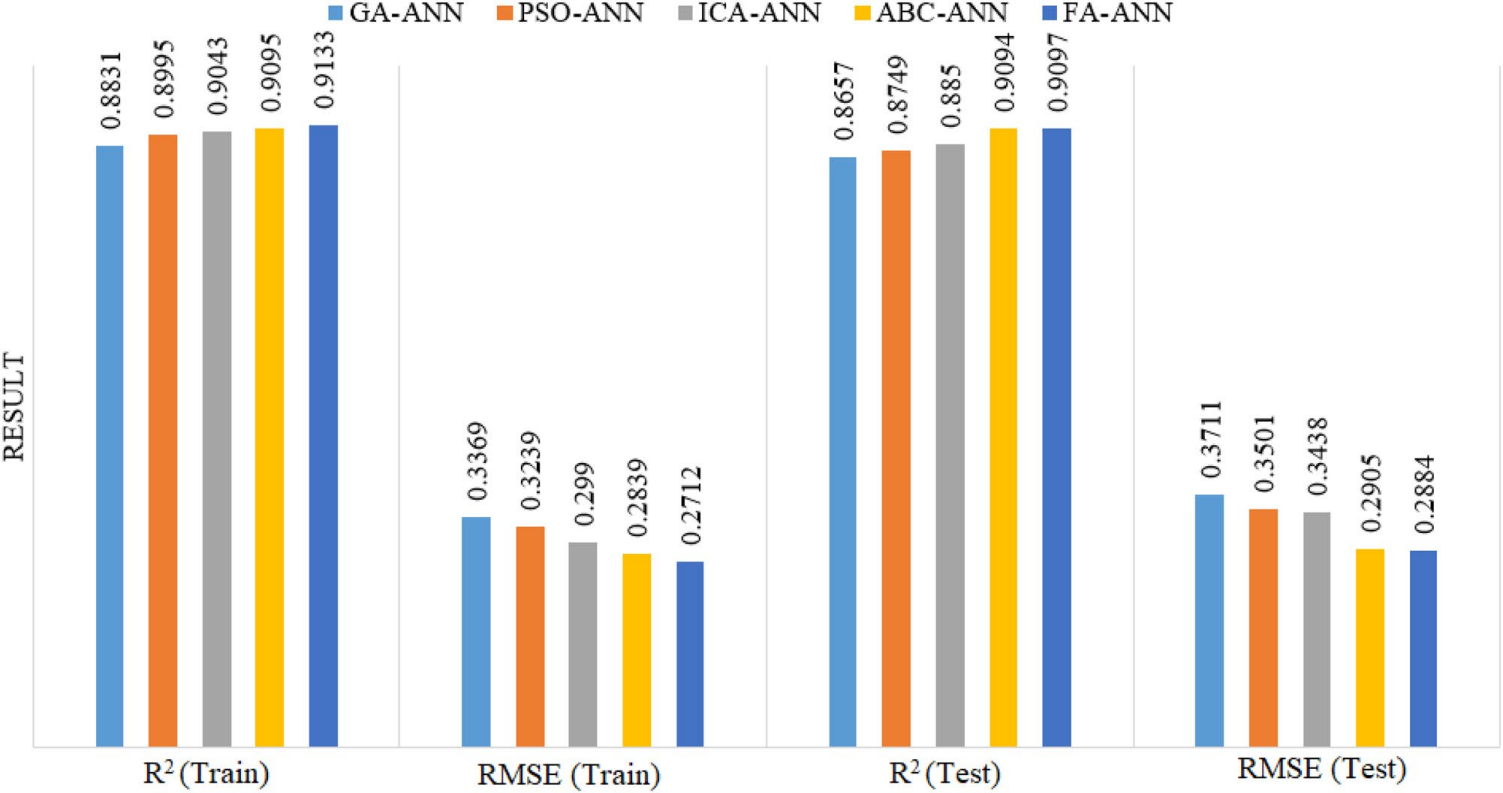
RMSE and R^2^ results of the selected hybrid models for prediction of PPV.

This study was mainly aimed to prediction PPV resulting from blasting in an almost accurate way. To this end, parameters with the highest influence upon the BIGV were determined. Five ANN-based models i.e., ICA-ANN, GA-ANN, ABC-ANN, FA-ANN and PSO-ANN, were taken into account to choose the best among for the prediction of PPV. For each of the predictive techniques, lots of hybrid models were configured among which the best one was selected. Figure 15 presents the results obtained from the chosen models of ICA-ANN, PSO-ANN, GA-ANN, ABC-ANN and FA-ANN on the basis of the R^2^ and RMSE indices for train and test phases. The great efficiency of the training datasets shows the success of the learning processes of the predictive models in cases where those of the testing datasets reflect the acceptable generalizability of the models. Therefore, the predictive model of FA-ANN was found capable of offering the capacity of a superior performance in regard to both R^2^ and RMSE values of both training and testing phases. R^2^ results of (0.8831, 0.8995, 0.9043, 0.9095 and 0.9133) and (0.8657, 0.8749, 0.885, 0.9094 and 0.9097) were obtained for train and test stages of GA-ANN, PSO-ANN, ICA-ANN, ABC-ANN and FA-ANN models, respectively. Moreover, RMSE values of (0.3369, 0.3239, 0.299, 0.2839 and 0.2712), (0.3711, 0.3501, 0.3438, 0.2905 and 0.2884) were obtained for train and test stages of GA-ANN, PSO-ANN, ICA-ANN, ABC-ANN and FA-ANN models, respectively. Among all, the FA-ANN model showed a higher level of prediction performance and lower level of system error. Although all hybrid models are able to predict PPV with acceptable performance prediction, FA-ANN is the best one among them and can provide higher prediction performance using only 4 input parameters.

**Figure 16:**
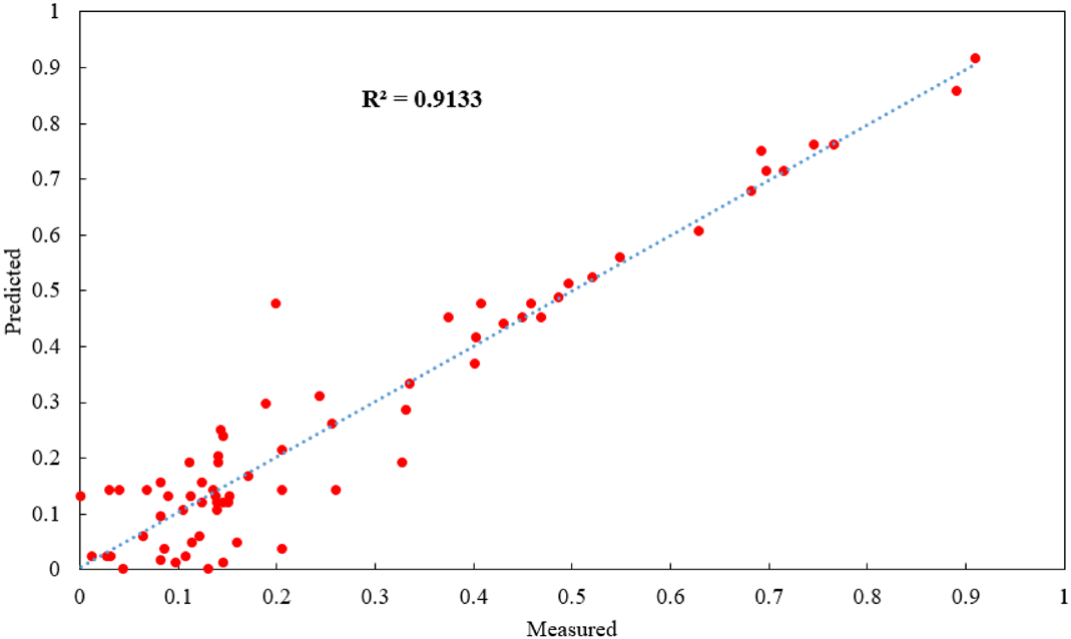
Training results of FA-ANN model in estimating PPV.

**Figure 17:**
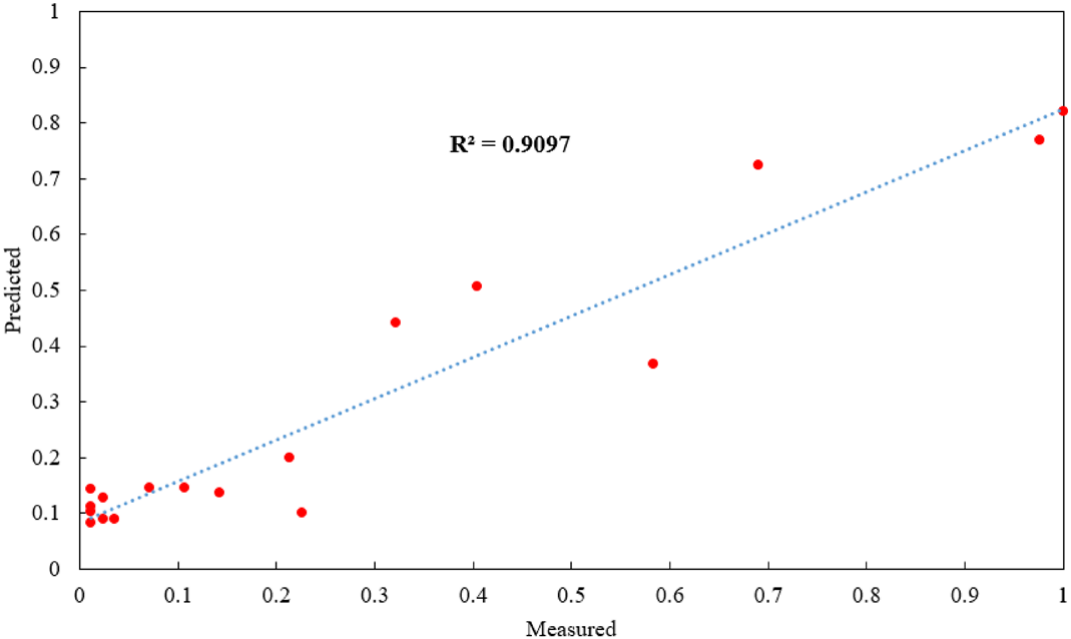
Testing results of FA-ANN model in estimating PPV.

As mentioned before, Hajihassani et al.^76^ used the same datasets with 9 model inputs i.e., HD, MC, BS, St, SD, PF, RQD, D, and No. H and developed a PSO-ANN model. They obtained R^2^ results of 0.89 for the best PSO-ANN model (both training and testing) with 9 model inputs. The aim of this study is to reduce number of model inputs to 4 (out of 9) and receive higher prediction performance compared to the previously published study. Results of this study according to R^2^ are 0.9133 and 0.9097 for training and testing datasets (see Figs. 16 and 17), respectively. It should be mentioned that a model with minimum No. of model inputs is always of interest and advantage. On the other hand, in case of utilize this model by other researchers and engineers, they need to collect only 4 parameters as inputs instead of 9 input parameters in the study conducted by Hajihassani et al.^76^. Therefore, the authors believe that the proposed FA-ANN model of this study is superior compared to previously PSO-ANN hybrid model.

## Conclusions

The results obtained from a total of 88 blasting events were considered to construct five hybrid intelligent models, i.e., ICA-ANN, PSO-ANN, GA-ANN, ABC-ANN and FA-ANN to predict PPV. Due to the importance of this issue for the environment, detailed operations were carried out during 6 months to prepare the database. For this purpose, 4 parameters (*MC*, *D*, *PF*, *SD*) out of 9 parameters (*HD*, *MC*, *BS*, *St*, *SD*, *PF*, *RQD*, *D*, and No. *H*) were selected as model inputs in a way to effectively predict the PPV values. This process was made through the use of FDM as one of the decision making techniques. It can be said that the first academic contribution of this study is referred to the use of expert’s opinions to reduce the total number of input parameters and determine the most effective ones on the ground vibration. The second academic contribution of this paper is related to a combination of different optimization algorithms with the ANN model aiming to optimize its weighs and biases to get the higher performance for PPV prediction. We attempted in this study to identify the parameters of the highest significance in the optimization algorithms i.e., ABC, ICA, GA, FA and PSO considering their backgrounds as well as existing literature. After that, the identified parameters were examined through conducting a number of parametric studies. The results were assessed by means of the simple ranking technique, which confirmed the high capability and accuracy of all hybrid models in the estimation of the PPV.

According to the obtained results, among all developed ANN-based models, the use of the FA-ANN model showed a marginally higher quality of performance in the predictive tasks (which was associated with the lower frequency of errors and a superior determination coefficient). RMSE values of (0.3369, 0.3239, 0.299, 0.2839 and 0.2712) and (0.3711, 0.3501, 0.3438, 0.2905 and 0.2884) were obtained in case of training and testing of the GA-ANN, PSO-ANN, ICA-ANN, ABC-ANN and FA-ANN predictive models, respectively. Findings confirmed that the FA-ANN model outperformed the other models considered in this study. In addition, a comparable trend was achieved in case of R^2^. Therefore, we came to the conclusion that where there is a need for a predictive model with the lowest level of error, the FA-ANN model can be applied efficiently to accurate prediction of the PPV resulting from blasting. The proposed models can be used in the other mines with granite rock type where the same input parameters with their ranges must be considered.

The main limitation of this study is the lack of a general model incorporating a wide range of input and output parameters. Further studies with the same model inputs and output and a larger number of data samples can be conducted proposing the same ANN-based models. In addition, since the accuracy of the ANN-based techniques is greatly affected by the use of different OAs, the performance capacity of these models can be improved by replacing some other newer optimization algorithms such as cat swarm, artificial fish swarm and gray wolf instead of the used OAs. They may then compare with the proposed models in this study for comparison purposes.
